# Psychological resilience and social support as determinants of HIV-related stress in older adults living with HIV in China: a moderated mediation analysis

**DOI:** 10.1186/s12889-025-25159-w

**Published:** 2025-11-10

**Authors:** Huang Cong, Mo Juan, Lei Xianyang, Amy Micaela Montufar Mejia, Dama Faniriantsoa Henrio Marcellin, Chen Zhong

**Affiliations:** 1 Department of Epidemiology and Health Statistics, Xiangya School of Public Health, Central South University, Changsha, Hunan 410013 China; 2 Medical Department, The First Hospital of Changsha, Changsha, Hunan 410000 China; 3 Department of Social Medicine and Health Management, Xiangya School of Public Health, Central South University, Changsha, Hunan 410013 China; 4 Department of Clinical Medicine , The third Xiangya Hospital, Central South University, Changsha, Hunan 410013 China; 5 Department of Anatomy and Neurobiology, School of Basic Medical Science, Central South University, Changsha, Hunan 410013 China; 6 Department of Infection and Immunology, The First Hospital of Changsha, Changsha, Hunan 410000 China

**Keywords:** Older adults living with HIV, Social support, Psychological resilience, HIV-related stress, Mediation, Moderation

## Abstract

**Background:**

This cross-sectional study investigates the relationship between social support, psychological resilience, and HIV-related stress in older adults living with HIV in China. It further explores whether psychological resilience mediates the link between social support and stress and whether living arrangements moderate this relationship.

**Methods:**

From January to February 2024, 405 older adults aged 50 and above were selected from the outpatient Department of Infection and Immunology at Changsha Infectious Disease Hospital. Participants filled out a structured on-site questionnaire survey measuring the Chinese HIV/AIDS Stress Scale (CSS-HIV), the Connor-Davidson Resilience Scale (CD-RISC-10), and the Social Support Rating Scale (SSRS). Data were analyzed using SPSS 26.0, GraphPad Prism 10, and the PROCESS macro to test for mediation and moderation effects.

**Results:**

The mean HIV-related stress score was 24.84 ± 14.01, comprising instrumental (4.71 ± 4.74), social (14.23 ± 6.21), and emotional (5.91 ± 5.98) stress components. Social support had a significant total effect on HIV-related stress (β = -0.462, *p* < 0.001). The indirect effect via psychological resilience was statistically significant (β = -0.216, 95% CI [-1.044, -0.339], *p* < 0.001), and the direct effect remained significant (β = -0.246, *p* = 0.025), indicating partial mediation. Living with others showed a stronger moderating effect on psychological resilience against stress (β = -1.004, *p* < 0.001) than living alone (β = -0.586, *p* < 0.001).

**Conclusion:**

In this study, we found that psychological resilience and social support play critical roles in reducing HIV-related stress among older adults living with HIV in China. Our findings suggest that interventions designed to strengthen resilience and promote a supportive social environment have the potential to significantly improve mental health outcomes for this population. Future programs should consider the role of living arrangements by addressing privacy challenges and the dynamics of shared living, and by recognizing how these factors shape stress experiences and support needs in this vulnerable population.

**Trial registration:**

Not applicable. This study is a cross-sectional observation study and was therefore not registered as a clinical trial.

## Introduction

In recent years, HIV/AIDS has remained a major global public health concern. The Joint United Nations Programme on HIV/AIDS (UNAIDS) estimates that by December 2022, about 39 million people were living with HIV (PLWH) globally, of whom 29.8 million were on antiretroviral therapy (ART). In the same year, 1.3 million new HIV cases were recorded, and 630,000 HIV-related deaths occurred [1, 2]. To address this burden, UNAIDS then established the “95-95-95” targets, aiming to eradicate HIV/AIDS as a public health threat by 2030 [3].

In China, the first AIDS case was documented in 1985 [4]. By late 2022, an estimated 1.22 million people were living with HIV [2, 5], increasing to around 1.329 million by mid-2024, with 474,000 HIV-related deaths reported [6]. As the pandemic spreads, the growing number of older adults living with HIV has become a major concern. Following the Chinese and US Centers for Disease Control and Prevention (CDC) [7–10], we define “older adults” in this study as individuals aged 50 or above, a common threshold in HIV research due to accelerated aging and earlier onset of age-related comorbidities, even though the World Health Organization (WHO) generally uses 60 or 65 as the threshold.

Stress levels in older adults living with HIV are influenced by a combination of internal psychological traits, such as personality and resilience [11, 12], and external social factors, such as stigma, discrimination, and support networks [13, 14]. Additionally, age-related physical decline, HIV-related stigma, social isolation, and mental health vulnerabilities [15, 16] can further lead to anxiety, depression, and, in certain situations, suicidal thoughts, all of which can significantly affect daily functioning and overall well-being [17, 18]. While previous studies [19, 20] (Chepkemoi, S., et al., and Tam, C., et al.) explored social support or psychological resilience separately, few have examined their interacting effects through a moderated mediation framework. The majority of existing studies have focused on younger adults or generalized groups [21, 22], which frequently leaves the experiences of this vulnerable population understudied. Thus, a significant gap exists in understanding the mechanisms (mediation) and boundary conditions (moderation) that link psychosocial resources to stress outcomes in this specific demographic.

Moreover, social support is a well-established protective factor against psychological suffering, such as stigma, anxiety, and depression [23]. An individual’s ability to positively react to adversity is known as psychological resilience, sometimes referred to as “psychological elasticity,” and it is crucial for treating long-term illnesses like HIV [24]. Many studies on the relationship between social support and stress in older adults living with HIV have not adequately considered psychological resilience as a potential mediating factor. However, resilience is critical for coping with chronic disease and fostering emotional well-being [24–26]. Recent evidence suggests that psychological resilience may mediate the relationship between social and mental health outcomes [24], yet this interaction has rarely been examined in older adults living with HIV.

To theoretically anchor these relationships, we use the Transactional Model of Stress and Coping [27]. This model conceptualizes stress as the result of an individual’s dynamic cognitive appraisal of external demands and their internal coping mechanisms. In this context, social support serves as a key external resource that can mitigate stress by positively influencing primary (threat assessment) and secondary (coping option assessment) appraisal processes, as well as aiding emotional regulation. Psychological resilience, conversely, represents a vital internal coping resource, particularly a set of traits that facilitate adaptive responses to adversity by enhancing cognitive flexibility and perseverance. We therefore hypothesize that social support reduces perceived HIV-related stress both directly and indirectly by bolstering psychological resilience (the mediating pathway).

Living arrangements may also play an important role in this dynamic, particularly within the Chinese cultural context. On one hand, cohabitation, which is common in China due to strong familial values and intergenerational living traditions, may enhance the availability of daily help and emotional support, thereby strengthening psychological resilience [28]. On the other hand, living alone may promote more independence and autonomy but can also increase vulnerability to social isolation and loneliness, and a deficit in the practical support critical for managing a chronic condition like HIV [15, 29–31]. However, both living arrangements present unique challenges; cohabitation may introduce stressors, such as loss of privacy, fear of HIV status disclosure, interpersonal conflict, or experiences of stigma within the household itself, while living alone may increase vulnerability to loneliness and limited access to immediate support [14, 31]. This complex, dual nature of living arrangements suggests that their effect is not uniform but likely depends on an individual’s personal resources. We propose that living arrangements act as a contextual moderator, specifically influencing the strength of the relationship between psychological resilience and stress. For individuals with high resilience, living with others may provide an environment to leverage that resilience effectively against stress. For those with low resilience, the potential stressors of cohabitation may overwhelm their coping capacity, thereby weakening the protective effect that psychological resilience typically has on stress. Despite its relevance, few studies have examined how living arrangements moderate the effects of social support and psychological resilience on stress, an area that remains underexplored in the existing literature and which this study aims to address.

This study aims to close these gaps by explicitly testing a moderated mediation model exploring the relationship between social support, psychological resilience, and HIV-related stress in older adults living with HIV in China. Consequently, we hypothesize that social support reduces HIV-related stress both directly and indirectly by strengthening psychological resilience (the mediating pathway). We further propose that this protective mechanism is contingent on context. Specifically, we expect living arrangements to moderate the relationship between resilience and stress, such that the benefit of high resilience is amplified for individuals living with others compared to those living alone. A more profound understanding of these psychosocial dynamics can inform targeted interventions to improve the mental well-being of this vulnerable population.

## Materials and methods

### Participants and data collection

From January to February 2024, this cross-sectional study was carried out at Changsha Infectious Disease Hospital’s HIV clinic, which is part of the Department of Infection and Immunity. This hospital was selected as the study site due to its status as a major regional center for HIV treatment, serving a large population of PLWH from across Hunan province, which ensured adequate participant recruitment. The patients were older adults with HIV who were 50 years of age or older. Those patients had to meet the following inclusion criteria: a confirmed HIV diagnosis from the CDC, sufficient cognitive and language abilities to understand and complete the questionnaires. Data collection occurred at a single point per participant, with no follow-up period. The primary exposures measured were the levels of social support and psychological resilience, and the primary outcome was perceived HIV-related stress. Severe cognitive impairment, diagnosed psychiatric conditions or language inability to understand and complete the questionnaires, were among the exclusion criteria. All questionnaires were checked for completeness at the time of data collection. Cases with missing data on key variables (social support, psychological resilience, or HIV-related stress) were excluded from analysis using listwise deletion. No imputation methods were applied. This study was approved by the Ethics Committee of Changsha First Hospital (Approval No.: KX-2021028) and conducted in accordance with the Declaration of Helsinki. All participants provided written informed consent before participation. They were informed of the study’s purpose, their right to refuse or withdraw at any time, and the confidentiality of their responses. No minors were involved in the study.

### Survey process and measures

Participants were recruited using a convenience sampling method. Investigators were trained in advance, and participants were recruited after being informed of the study’s purpose and providing consent. One-on-one surveys were conducted in a separate room using a self-developed questionnaire covering the following sections:

1. Demographic information: collected based on medical records: gender, age, marital status, ethnicity, education, household registration, work status, diagnosis time, comorbidities, most recent CD4 count [32], and living arrangement, which was divided into two categories and was recorded to assess whether participants were living alone or with others. For analysis, this variable was recoded as a binary indicator: 0 = living alone and 1 = living with others (spouse, children or parents, friends, etc.). This recording ensured that living alone served as the reference group, allowing for direct comparison with participants cohabiting with others.
2. HIV-related stress: The three dimensions of stress—social, emotional, and instrumental—were measured using the Chinese version of the HIV/AIDS-related stress scale (CSS-HIV), revised by Niu Lu et al. [32]. We used the 5-point Likert scale, which ranges from 0 to 68 points. A higher score reflects greater stress related to HIV. The study’s Cronbach’s *α* value was 0.900, which is the internal consistency coefficient.
3. Psychological resilience: Assessed using the 10-item Connor-Davidson Resilience Scale (CD-RISC-10). It consists of 10 items scored with a 5-point Likert scale ranging from 0 to 40 points. This assessment was done to find out how resilient the patients were psychologically [33]. A higher score indicates stronger resilience. This study’s internal consistency coefficient was Cronbach’s *α* = 0.951.
4. Social Support: The Social Support Rating Scale (SSRS) was used to measure social support by revising the method of Xiao et al., [34], which consists of 10 items in three dimensions: objective support, subjective support, and social support utilization. Patients with higher scores indicate greater support. This internal consistency coefficient for this study was Cronbach’s *α* = 0.704.

### Statistical methods

This study follows the STROBE reporting guideline. Excel was used for preliminary data processing and is available from the corresponding author upon reasonable request due to privacy restrictions. GraphPad Prism 10 and SPSS 26.0 were used for the common method bias testing, group comparisons, descriptive statistics, and correlation analysis. Moderation (PROCESS Model 58) and mediation (PROCESS Model 4) analyses were conducted using the PROCESS 3.4 plug-in. In this analysis, the moderator variable living arrangement was coded as 0 = living alone and 1 = living with others to test whether cohabitation buffered the impact of psychological resilience on HIV-related stress. This coding approach aligns with the theoretical expectation that cohabitation is associated with increased social support and lower stress. Statistical significance was set at *p* < 0.05. Quantitative variables, such as social support, psychological resilience, HIV-related stress scores, age, and CD4 count, were treated as continuous variables. This study was not preregistered, and no analysis code or materials are publicly archived due to the proprietary nature of the instruments used. Given the convenience sampling strategy, no weighting adjustments or complex survey design corrections were applied; thus, results should be interpreted with caution regarding representativeness.

## Results

### Basic information about the participants

During this study, a total of 450 older adults living with HIV were approached for participation, and 27 declined due to personal reasons (lack of interest, privacy concerns, and time constraints). Among the 423 eligible participants, 18 individuals were excluded due to incomplete questionnaires, communication barriers, or withdrawal of consent, resulting in a final sample of 405. The mean age was 59.34 ± 7.0 years, with an average diagnosis time of 5.44 ± 4.08 years. The majority of the participants were male (69.10%), married or cohabiting (71.40%), urban permanent residents (54.32%), and of Han ethnicity (98.77%). Most participants had no comorbidities (58.02%) and were in stage I (HIV-1) of the disease (44.20%). The majority of the participants mainly lived with others (77.78%) (Table 1).

**Table 1:** Participant demographic and clinical characteristics (n=405)

Characteristics	Values (%) or mean ± SD
Total subject	405
Average age (Years)	59.34 ± 7.0
Average diagnosis time (Years)	5.44 ± 4.08
Gender (Male)	69.10%
Marital status (Married/cohabiting)	71.40%
Residency (Urban resident)	54.32%
Ethnicity (Han)	98.77%
Comorbidities (No comorbidities)	58.02%
Disease stage (Stage I, HIV-1)	44.20%
Living arrangements (Living with others)	77.78%

### Common method deviation

The outcomes of Harman’s single-factor test indicated 7 factors with eigenvalues ​​greater than 1. The first factor accounted for 31.07% of the variance, falling below the threshold of 40%. This suggests that the common method bias had relatively no effect on this study.

### Social support, psychological resilience, and HIV-related stress scores

The study examined social support, psychological resilience, and HIV-related stress in 405 older adults living with HIV, with no missing data for the key variables. The mean social support score was 36.34 ± 7.39 (range 0–66), psychological resilience was 28.22 ± 7.67 (range 0–40), and the total HIV-related stress score was 24.84 ± 14.0 (range 0–68). Scores for stress dimension showed emotional stress: 5.91 ± 5.98, social stress: 4.23 ± 6.21, and instrumental stress: 4.71 ± 4.74 (each reported on its original scale) **(**Table 2**).** These descriptive results highlight variability in stress experiences and coping resources among participants. Each construct is reported on its original measurement scale to avoid misleading cross-scale comparisons.

**Table 2:** Descriptive statistics for the study variables, reported on their respective scales

Variable	Mean (M)	Standard deviation (± SD)	Score range
Social support (SSRS)	36.34	7.39	0–66
Psychological resilience (CD-RISC-10)	28.22	7.67	0–40
Total HIV-related stress (CSS-HIV)	24.84	14.0	0–68
Emotional stress dimension	5.91	5.98	0–20
Social stress dimension	14.23	6.21	0–20
Instrumental stress dimension	4.71	4.74	0–20

### Correlation analysis between social support and psychological resilience, and HIV-related stress

As shown in the Pearson correlation analysis, social support was significantly positively associated with psychological resilience (*r* = 0.225, *p* < 0.01). Additionally, social support showed a strong negative correlation with HIV-related stress (*r* =−0.537, *p* < 0.01), while psychological resilience also showed a significant negative correlation with HIV-related stress (*r* =−0.244, *p* < 0.01**) **(Table 3). These correlations support the theoretical basis for the mediation and moderation analyses.

**Table 3:** Pearson correlation among dimensions of social support, psychological resilience, and HIV-related stress (*r-*value)

Variable	Social support	Psychological resilience	Emotional stress	Social stress	Instrumental stress	Total HIV-related stress
Social support	——					
Psychological resilience	0.225**	——				
Emotional stress	−0.592**	−0.177**	——			
Social stress	−0.267**	−0.207**	0.458^**^	——		
Instrumental stress	−0.491**	−0.227**	0.685^**^	0.456^**^	——	
HIV-related stress	−0.537**	−0.244**	0.861^**^	0.792^**^	0.833^**^	——

*SSRS* Social Support Rating Scale, *CD-RISC-10* Connor-Davidson Resilience Scale-10, *CSS-HIV* Chinese HIV/AIDS Stress Scale

^**^p < 0.01

### The indirect effect of psychological resilience (mediator) between social support and HIV-related stress

The analysis revealed that social support had a statistically significant negative effect on HIV-related stress (ꞵ=−0.537, t=−12.78, *p* < 0.001), explaining 28.8% of the variance (R^2^ = 0.288, F (1,403) = 163.01). When psychological resilience was added as a mediator, the direct effect of social support on stress remained statistically significant (ꞵ=−0.246, t=−3.045, *p* = 0.025), showing partial mediation. Social support also significantly predicted psychological resilience (ꞵ=0.225, t = 4.63, *p* < 0.001), which in turn had a significant negative impact on stress (ꞵ=−0.928, t=−11.890, *p* < 0.001) **(**Table 4**)**. These findings support the presence of a statistically significant partial mediation effect (a significant indirect effect).

**Table 4:** The test of the indirect effect of resilience between social support and HIV-related stress

Outcome variable	Predictor variable	Fit indicators				Coefficient significance	
		*r*	*R* ^2^	F	β	t	*p*
HIV-related stress	Social support	−0.537	0.288	163.01	−0.537	−12.78	< 0.001**
Psychological resilience	Social support	0.225	0.051	21.66	0.225	4.63	< 0.001**
HIV-related stress	Social support	0.552	0.304	176.02	−0.246	−3.045	0.025*
	Psychological resilience				−0.928	−11.890	< 0.001**

Regression results for the mediation analysis

^*^p < 0.05.^**^p < 0.001

Bootstrapped confidence intervals (CI) confirmed that both the direct and indirect (mediating) effects were significant: the 95% CI for the direct effect was [−0.405, −0.087], and for the indirect effect of psychological resilience, [−1.044, −0.339]. The total effect of social support on stress (ꞵ= −0.462) was decomposed into a direct effect (ꞵ=−0.246, 53.25%) and an indirect effect via psychological resilience (ꞵ= −0.216, 46.75%) (Table 5). All estimates presented are unadjusted or adjusted only for variables specified in the hypothesized models. No additional confounders were statistically controlled for, as the primary focus was on psychological pathways rather than demographic predictors.

**Table 5:** Decomposition table of total effect, direct effect, and indirect effect

Effect type	Effect value	SE	95% CI	Effect proportion	*P*-value
Indirect effect	−0.216	0.059	[−1.044~−0.339]	46.75%	< 0.001**
Direct effect	−0.246	0.081	[−0.405~−0.087]	53.25%	0.025*
Total effect	−0.462	0.092	[−0.642~−0.283]	100%	< 0.001**

Decomposition direct, indirect, and total effects

^*^p < 0.05.^**^p < 0.001

### The moderating effect of living arrangements (as a moderator)

We tested the moderating effect of living arrangements on the mediation model (Table 6). The interaction between psychological resilience and living arrangements had a significant negative effect on HIV-related stress (β=−0.418, *p* = 0.022), indicating that living arrangements moderate the relationship between psychological resilience and stress. The full model, including the interaction term, explained a significant proportion of variance in stress (R^2^ = 0.321). Specifically, psychological resilience reduced HIV-related stress in both groups, but this protective effect was stronger among individuals living with others (β= −1.004, *p* < 0.001) compared to those living alone (β= −0.585, *p* < 0.001). This difference in slopes was statistically significant, as shown by the interaction term (β=−0.418, *p* = 0.022), suggesting that cohabitation enhances the stress-buffering role of psychological resilience in older adults living with HIV. A hierarchical regression analysis was used: first testing the main effect of psychological resilience on stress, then the independent effect of living arrangements, and finally the interaction term (psychological resilience × living arrangement).

**Table 6:** Regression analysis of the moderating effect of living arrangements

Regression equation		Fit Indicator			Regression coefficient		
Outcome variable	Predictor variable	*r*	*R* ^*2*^	*F*	*β*	*t*	*p*
HIV-related Stress	Psychological resilience (living alone = 0)				−0.585	−3.608	< 0.001**
	Psychological resilience (living with others = 1)				−1.004	−6.482	< 0.001**
	Psychological resilience × Living situation	0.567	0.321	47.308	−0.418	−2.300	0.022*

Results of the moderation analysis

^*^p < 0.05.^**^p < 0.001

### Stratified regression analysis of psychological resilience on HIV-related stress by living arrangements

**Fig. 1:**
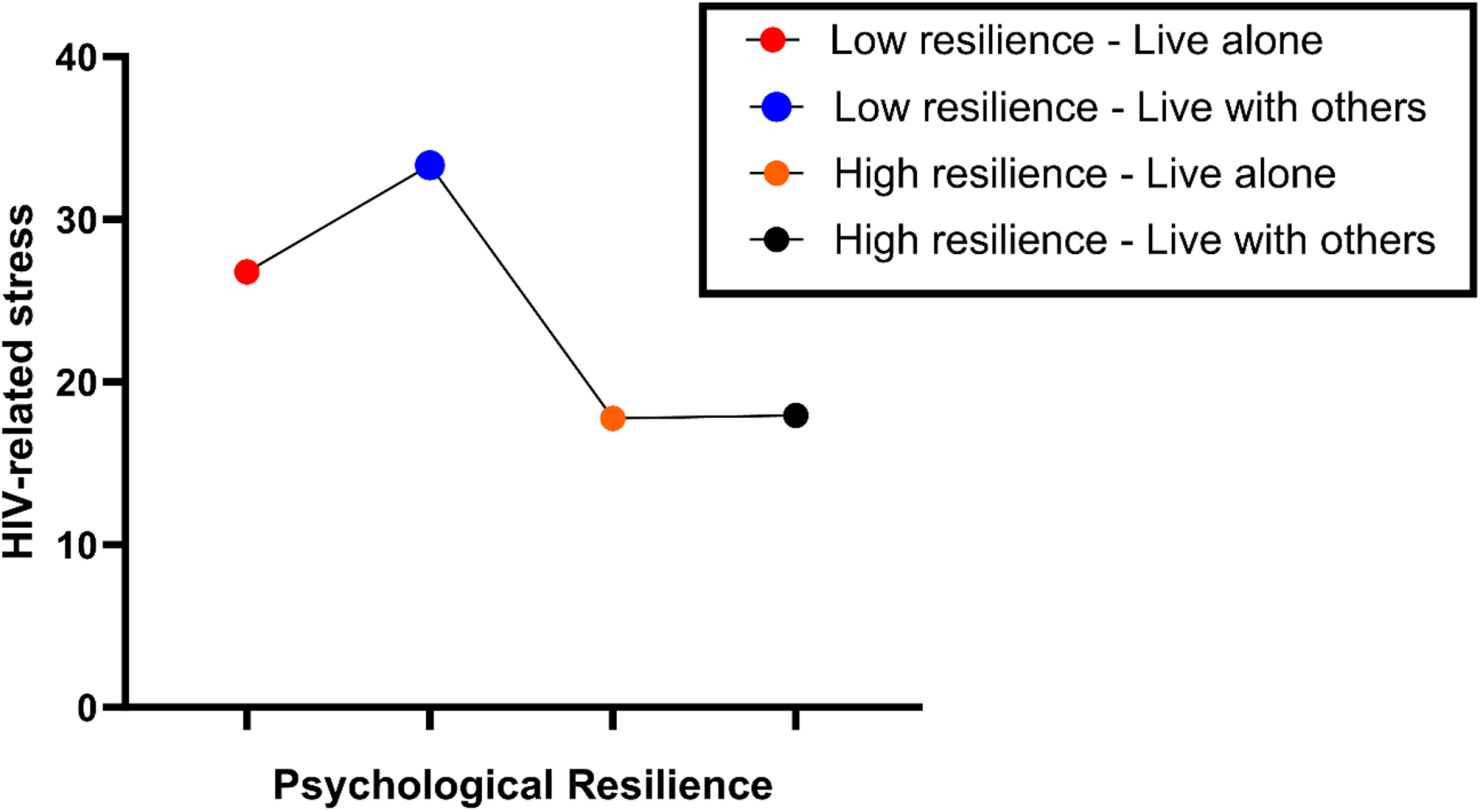
Moderating effect of living arrangements on the relationship between psychological resilience and HIV-related stress. Colors indicate groups based on living arrangement and resilience level. The scatter slope for participants living with others demonstrates the enhanced protective effect of psychological resilience in this group

To further investigate the moderating role of living arrangements, we performed a stratified regression analysis to assess the effect of psychological resilience on HIV-related stress in individuals living alone versus those living with others (Table 6 and Fig. 1). While no sensitivity analyses were conducted, results showed that psychological resilience significantly reduces HIV-related stress in both groups, with a stronger effect among those living with others (β =−1.004, *p* < 0.001) than those living alone (β =−0.585, *p* < 0.001). These findings indicate that shared living environments may enhance the protective role of psychological resilience. Nonetheless, individuals in cohabitating arrangement may also experience stressors, such as privacy concerns and interpersonal conflicts, which could explain the relationship between resilience and perceived stress in older adults living with HIV.

### Visual representation of the moderating effect

To further understand this moderating effect, Fig. 1 shows the moderating effect of living arrangements on the relationship between psychological resilience and HIV-related stress. The interaction term is significant (β=−0.418, *p* < 0.05) **(**Table 6**)**, indicating that living with others enhances the stress-buffering effect of psychological resilience.

## Discussion

This study assessed HIV-related stress in older adults living with HIV and its correlation with social support and psychological resilience. The average HIV-related stress score was 24.84 ± 14.01, which was higher than the scores reported in previous findings by Huang Yunxiang et al. [35], and this increase is primarily attributed to social stress factors, which is consistent with earlier studies [35, 36]. In China, stigma and discrimination against older adults living with HIV remain a major societal phenomenon [16, 37–39], which intensifies their psychological suffering. In addition, physical challenges, including declining physiological functioning, limited information access, and dwindling social networks, significantly impact this condition. Their social life may become even more difficult and stressful as a result of these problems.

Our findings show that social support reduces HIV-related stress indirectly through psychological resilience, with this indirect effect accounting for 46.75% of the total effect. This aligns with previous research indicating that higher social support is associated with lower stress, both directly and indirectly via resilience [24]. Similarly, findings by Ji Jiahao et al. et al. and Miaomiao Zhao et al. [40, 41] showed that resilience is essential for converting social support into real benefits for mental health. However, the indirect effect was moderate, suggesting that other variables, like stigma and financial limitations, may also contribute to and influence stress.

We also observed a significant negative correlation emerge between social support, psychological resilience, and stress. Individuals with higher social support are typically associated with stronger psychological resilience, which is known to foster self-esteem and a sense of community and enhance confidence [24]. Those with higher levels of psychological resilience cope more effectively with adversity, which further reinforces their support networks. Previous studies have shown that adequate social support not only helps reduce stress but also improves both physical and mental health outcomes in older adults living with HIV [42]. These findings emphasize the necessity of building both psychological resilience and social support to effectively reduce stress. Increasing patients’ social support through targeted health education and psychological counseling can further enhance their psychological resilience and, in turn, alleviate stress.

Besides the mediation effect, we explored how living arrangements influence the relationship between psychological resilience and perceived stress. Our results demonstrated that the protective effect of psychological resilience on stress was stronger for individuals living with others than for those living alone, as indicated by a larger negative regression coefficient (β =−1.004 vs. β =−0.585) and a model explaining a substantial portion of the variance in stress (R^2^= 0.321). This is consistent with the findings of Fang, X., et al., and Vorobyova, A [12, 43]., which show that social networks reinforce coping capacity. Cohabitating may, however, bring extra stressors, including privacy invasion or interpersonal conflicts [24, 44]. Despite this, our findings suggest that the effect of cohabitation on stress is complex, sometimes beneficial, sometimes harmful, depending on resilience levels. Living arrangements significantly influence the relationship between psychological resilience and perceived stress in this vulnerable population.

Interestingly, when resilience was low, individuals living with others reported feeling more stress than those who lived alone. Several factors may contribute to this, such as the burden of illness on family members, feelings of guilt or dependency, and issues related to personal space or privacy [45]. Intergenerational conflicts, especially with children, can further exacerbate psychological strain and increase daily life pressures [46]. Cohabitation can offer emotional and instrumental support but may also increase interpersonal conflicts and stigma [47]. For instance, younger family members may stigmatize, judge, or misunderstand older adults living with HIV, which can increase instead of reduce stress [48]. In some cases, the burden of illness may lead to feelings of guilt or dependency, further complicating family dynamics. Our results emphasize the importance of considering not only whether an individual lives with others, but also the quality of social interactions, the nature of relationships, and the household environment. Notably, as resilience improved, perceived stress significantly declined across both groups [49].

Public health programs aimed at improving mental well-being in older adults living with HIV should be tailored to both their living arrangements and levels of psychological resilience [13]. These programs should integrate psychological resilience training and community-based social support into HIV care [24]. Tailored services, such as peer-led support groups, family counseling, and culturally sensitive health education, can help enhance resilience and reduce stress [28]. Moreover, housing policies and aging-in-place initiatives should consider the psychological dynamics of shared living environments, ensuring that privacy and emotional well-being are protected [44, 50]. These targeted strategies may help reduce HIV-related stress and not only support national and China’s broader efforts but also contribute to achieving the UNAIDS 95-95-95 targets by 2030 [3].

The findings can be understood through the lens of the Transactional Model of Stress and Coping [27], in which psychological resilience functions as an internal coping mechanism that mediates the relationship between external resources and perceived stress. Based on the resilience theory, individuals who are more resilient are better able to maintain healthy emotional states and cognitively reframe stressors. Moreover, from the perspective of ecological models of behavior and health, the moderating effect of living arrangements reflects how contextual and social environments interact with internal traits to shape stress responses.

This study does, however, have several limitations. The data were collected using self-reported measures, which might have resulted in response bias due to social desirability or recall limitations [51]. Even the scales employed are validated; self-perceptions of stress, support, and resilience may not accurately reflect actual experiences. Future studies should include more objective measures, such as behavioral observations, clinician assessments, and physiological indicators. The cross-sectional design limits causal interpretation [52]. Participants were recruited from a single urban hospital using convenience sampling, which may limit generalizability to broader or rural populations. Cultural norms, healthcare access, and social support systems can vary regionally and internationally, which may influence the applicability [53]. Further research considering more diverse geographic and demographic samples is needed to enhance external validity. While the sample size was adequate, rural representation was limited in this study. Cross-cultural or multi-site studies, especially in low-resource settings, are recommended to validate these findings. Additionally, this study did not include demographic and clinical covariates (e.g., age, comorbidities, disease stage) in the mediation and moderation models. Although preliminary analyses showed no strong associations between these factors and stress outcomes, future research with larger, more diverse samples should explicitly adjust for such covariates to confirm the robustness of psychological pathways. Finally, this study excluded other potential moderators, including economic status and stigma. Longitudinal and qualitative research should be used to provide a deeper understanding of how social relationships and support systems shape stress experiences in older adults living with HIV. These limitations should be considered when interpreting the findings. The cross-sectional design precludes causal inferences, and the use of a convenience sample from a single urban clinic may limit the generalizability of the results to older adults living HIV in other settings, especially in rural areas.

As researchers from different backgrounds, we know how our identities and training may shape our interpretation of psychological resilience and social support. We approached this study with an awareness of potential biases and sought to emphasize participant perspectives through culturally sensitive data collection and analysis.

## Conclusion

In this study, we found that psychological resilience and social support play critical roles in reducing HIV-related stress among older adults living with HIV in China. Our findings suggest that interventions designed to strengthen resilience and promote a supportive social environment have the potential to significantly improve mental health outcomes for this population. Future programs should consider the role of living arrangements by addressing privacy challenges and the dynamics of shared living, and by recognizing how these factors shape stress experiences and support needs in this vulnerable population.

## Data Availability

The data that support the findings of this study are available on request from the corresponding author. The data are not publicly available due to privacy or ethical restrictions.

## Abbreviations

UNAIDS: Joint united nations programme on HIV/AIDS
CDC: Centers for disease control and prevention
WHO: World health organization
CSS-HIV: Chinese HIV/AIDS stress scale
CD-RISC-10: Connor-davidson resilience scale-10
SSRS: Social support rating scale
HIV: Human immunodeficiency virus
AIDS: Acquired immunodeficiency syndrome
PLWH: People living with HIV
ART: Antiretroviral therapy
SPSS: Statistical package for the social sciences
CI: Confidence interval
SE: Standard error
R^2^: Coefficient of determination (r-squared)
β (Beta): Regression coefficient
p: * p*-value (statistical significance level)
